# A Sustainable Translational Sheep Model for Planned Cesarean Delivery of Contraction-Free Ewes

**DOI:** 10.1007/s43032-023-01365-y

**Published:** 2023-10-17

**Authors:** Alexander Paping, Loreen Ehrlich, Kerstin Melchior, Thomas Ziska, Wolf Wippermann, Alexander Starke, Karin Heinichen, Wolfgang Henrich, Thorsten Braun

**Affiliations:** 1 Charité – Universitätsmedizin Berlin, corporate member of Freie Universität Berlin and Humboldt-Universität zu Berlin, Department of Obstetrics, Augustenburger Platz 1, 13353 Berlin, Germany; 2 Charité – Universitätsmedizin Berlin, corporate member of Freie Universität Berlin and Humboldt-Universität zu Berlin, Division of ‘Experimental Obstetrics’, Berlin, Germany; 3 Clinic for Ruminants and Swine, Faculty of Veterinary Medicine, Leipzig University, Leipzig, Germany; 4 Oberholz Farm for Teaching and Research, Faculty of Veterinary Medicine, Leipzig University, Leipzig, Germany

**Keywords:** Cesarean section, Basic research, Hysterotomy closure, Uterine sutures, Animal model, Animal welfare

## Abstract

We evaluated whether the sheep constitutes a useful translational model to evaluate anatomical and surgical aspects of cesarean delivery (CD) from a human medical perspective with the aim of both maternal and neonatal well-being. Our hypothesis was that CD in contraction-free ewes is not associated with major complications. Primary endpoint was the transferability of anatomical conditions and surgical techniques of CD from the ewe to the human. Secondary endpoints were maternal and fetal survival, occurrence of retained fetal membranes, metritis, mastitis, or wound infections. Forty-eight Merino ewes were delivered by CD after 95% gestation (142–144 days). Both ewes and newborn lambs were cared for intensively after the delivery. Ovine uterine anatomy during CD appeared slightly different but comparable to the human uterus. Uterine incisions were mostly performed in the uterine horns, not in the uterine corpus. The ovine uterine wall is thinner than in humans. All ewes survived without any major complications. Seventy-seven (88.5%) out of 87 live-born lambs survived without any complications. The contraction-free ewe constitutes an appropriate and safe model to evaluate anatomical and surgical aspects of CD from a human medical perspective. We present a step-by-step manual for successfully planned cesarean delivery for sheep including the perioperative management illustrated with photographs and a five-minute video. With adequate planning and a reasonable number of staff, it is possible to safeguard both maternal and neonatal survival. This sustainable translational medicine model offers additional potential for the offspring to be used for further research studies (e.g., transgenerational inheritance research).

**Supplementary Information:**

The online version contains supplementary material available at 10.1007/s43032-023-01365-y.

## Introduction

Animal models represent an alternative way to answer research questions that may be limited by ethical as well as patient concerns. The sheep is a long-used model for obstetric and gynecological research questions [1, 2]. Established translational research fields include antenatal corticosteroid treatment, fetal surgery, and uterine transplantation as well as pregnancy-related conditions such as preterm birth or placental insufficiency [3–8]. However, to date, no publications are available that discuss the suitability of the sheep model for cesarean delivery (CD) in terms of anatomical conditions, surgical, and practical feasibility. In particular, uterine wound healing has mainly been investigated in rodent models [9, 10]. We hypothesized that in this large animal model, surgery techniques can be performed similarly to those used in humans, making it easier to transfer research results. Due to their size, the ovine uterine corpus and horns can be easily distinguished. Studies on suturing techniques can be performed with suture thicknesses similar to those used in human surgery. While histological aspects of the female genital tract and in particular the structure of the uterine wall in sheep correspond to the structure in humans [11, 12], it remains to be clarified, in how far ovine uterine anatomy is comparable to humans. Litter size in sheep is much smaller than in rodents or pigs. Usually, a maximum of three lambs are born at a time. Therefore, adequate neonatal care can be ensured with a reasonable number of staff. Due to a lot of veterinary experience in neonatal lamb care, animal welfare can be maintained. Recently, a small study on eight sheep has investigated uterine scarring after CD using histological staining and biomechanical tests [13]. It therefore seems intriguing to establish the sheep as a model for uterine scarring post-CD. As CD is an operation that both the ewe and the lambs can survive, contemporary ethical and animal welfare considerations—such as the 3R principle—demand an appropriate study design that promotes maternal as well as neonatal well-being [14]. However, information on these topics is scarce, as it is still a common practice to euthanize both ewes and lambs after experiments [15, 16]. Existing veterinary publications deal with the technique of performing CD under field conditions [17–21] and focus on emergency CD of ewes under labor due to complications such as failure of the cervix to dilate, fetal-maternal disproportion, or fetal malpresentation [20, 22]. For standardized basic research, CD must be planned and therefore the uterus is contraction-free. This leads to the following unanswered questions: As the ovine placenta cannot be removed during CD, will it be born post-operatively? When? How many ewes will suffer from metritis due to retained fetal membranes? Will uterine atony cause excessive blood loss? Will the ewes survive? Which protocols are needed to prevent complications? Will the ewes accept their lambs? How are the lamb survival rates? Will the ewes start producing milk although they had no natural birth? If yes, will they suffer from mastitis? As these questions do not arise during veterinary practice, information is very scarce. We therefore established an animal-friendly sheep model for planned CD and evaluated surgical and anesthesiologic techniques with respect to maternal and neonatal outcome.

## Methods

### Study Design and Experimental Animals

Merino ewes (*N* = 48) were mated naturally. A search ram was let into the flock to locate the sheep currently in heat. A breeding ram individually mated the selected sheep. Mating was done according to a precise schedule, whereby we assumed that operations should be performed from Monday to Friday at 142 days of gestation (dG) or later, with a maximum of three CD per day. Seventeen ewes mated outside their usual oestrus season received prior oestrus synchronization with intravaginal application of 20 mg flugestone acetate (Chronogest CR, MSD, Rahway, USA) followed by 500 IU intramuscular PMSG (Pregmagon, Ceva Santé Animal, Libourne, France). Pregnancies were confirmed with transabdominal ultrasound at 52–60 dG. Only animals testing negative for the following pathogens prior to study inclusion: *Visna-maedi virus*, *Yersinia pseudotuberculosis*, *Chlamydia abortus*, *Coxiella burnetii*, *Bovine viral diarrhea virus*, *Brucella*, and *Mycoplasma* in serum were included. CD was performed at 142 dG or later as for lambs born at 95% of gestation or later, only mild prematurity, and therefore a mostly uncomplicated course, can be assumed [23]. The study protocol was approved by the country directorate of Saxony (Landesdirektion Sachsen, TVV 03/22).

### Preoperative Preparation

All ewes received dexamethasone (Dechra Veterinary Products, Aulendorf, Germany) on the day before CD to enhance fetal lung maturation and as a mild means of labor induction with the aim to support timely birth of the placenta and better acceptance of the lambs [24]. The first 12 ewes received only 6 mg dexamethasone subcutaneously. After this had proven to be without any risk of preterm delivery, the remaining 36 ewes received 10 mg dexamethasone.

### Anesthesia and Perioperative Medication

**Fig. 1:**
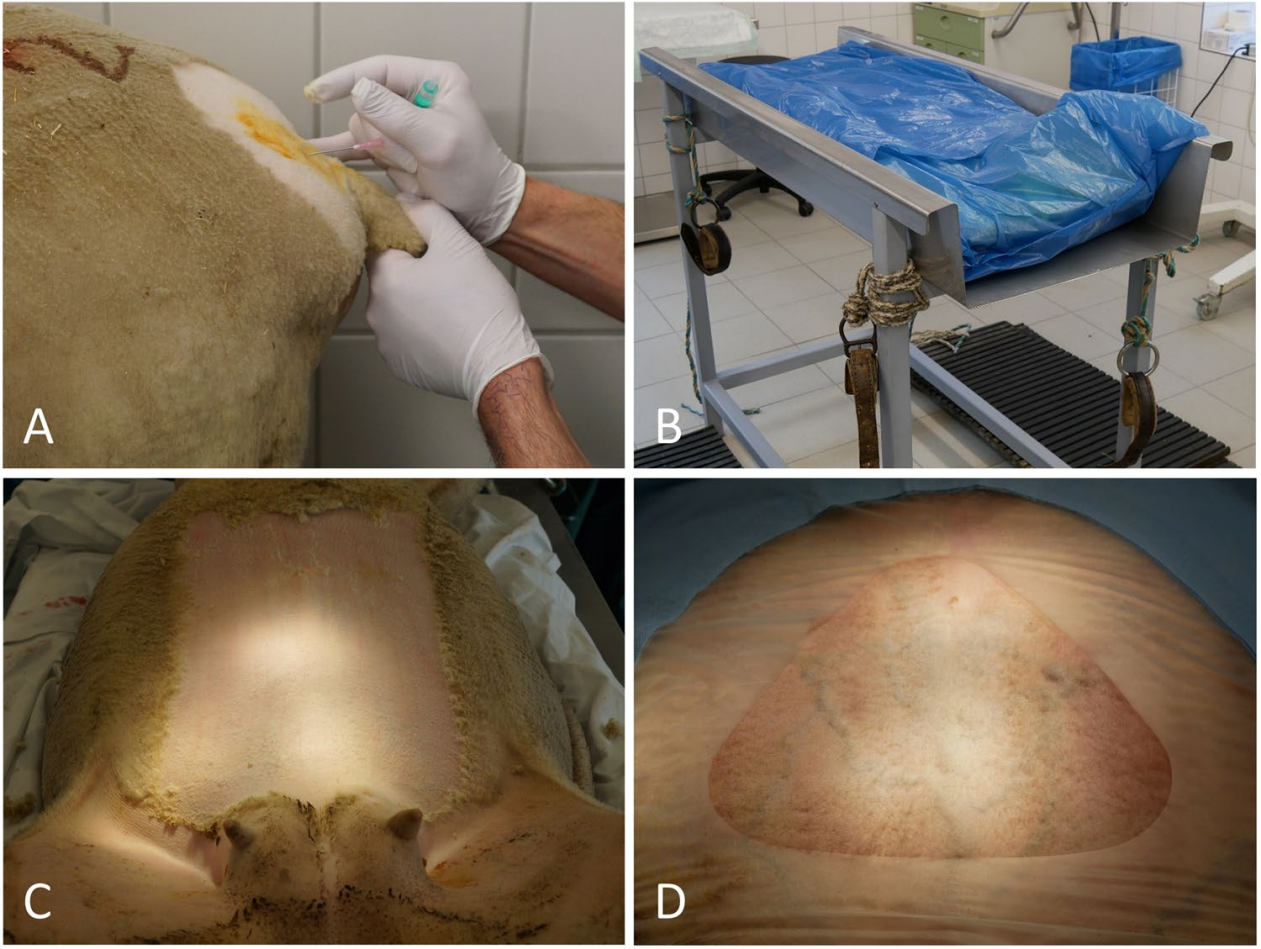
Preoperative preparations for cesarean delivery. **A** Sacrococcygeal epidural anesthesia with xylazine. **B** Operation table with high sides to prevent dislocation of the patient. Ropes are used to fix the extremities during the operation. **C** Sheep on the operation table in a supine position. Fur in the operating field has been shorn. **D** Operating field before line block. Note the dense net of subcutaneous veins draining blood from the udder. The area for paramedian entry into the abdomen is chosen such that the vessels are spared as much as possible

Epidural anesthesia with xylazine was combined with incision line infiltration as multimodal pain management under preoperative NSAID administration [25, 26]. Sacrococcygeal epidural anesthesia was performed with 0.2 mg/kg xylazine and 0.3 ml/kg 0.9% sodium hydrochloride (Figure 1A) and intravenous ketamine 200 mg. After delivery of the lambs, anesthesia was deepened with 0.1–0.3 mg/kg IV xylazine. After placing the ewe on the operating table, a catheter was inserted in the jugular vein. All ewes received 10 mg/kg IV amoxicillin (Zoetis Schweiz GmbH, Delémont, Suisse), 0.5 mg/kg IV meloxicam (Ceva Santé Animal), and 3000 IU IM anti-tetanus serum (MSD Animal Health GmbH, Lucerne, Suisse). To aid uterine contraction, oxytocin (Serumwerk Bernburg, Bernburg, Germany) was applied intravenously after delivery of the lambs (3 IU single shot + 10 IU in 500 ml 0.9% sodium hydrochloride). Cloprostenol at 0.125 mg was administered IM to facilitate uterine involution (Veyx-Pharma, Schwarzenborn, Germany).

### Operation Protocol

**Fig. 2:**
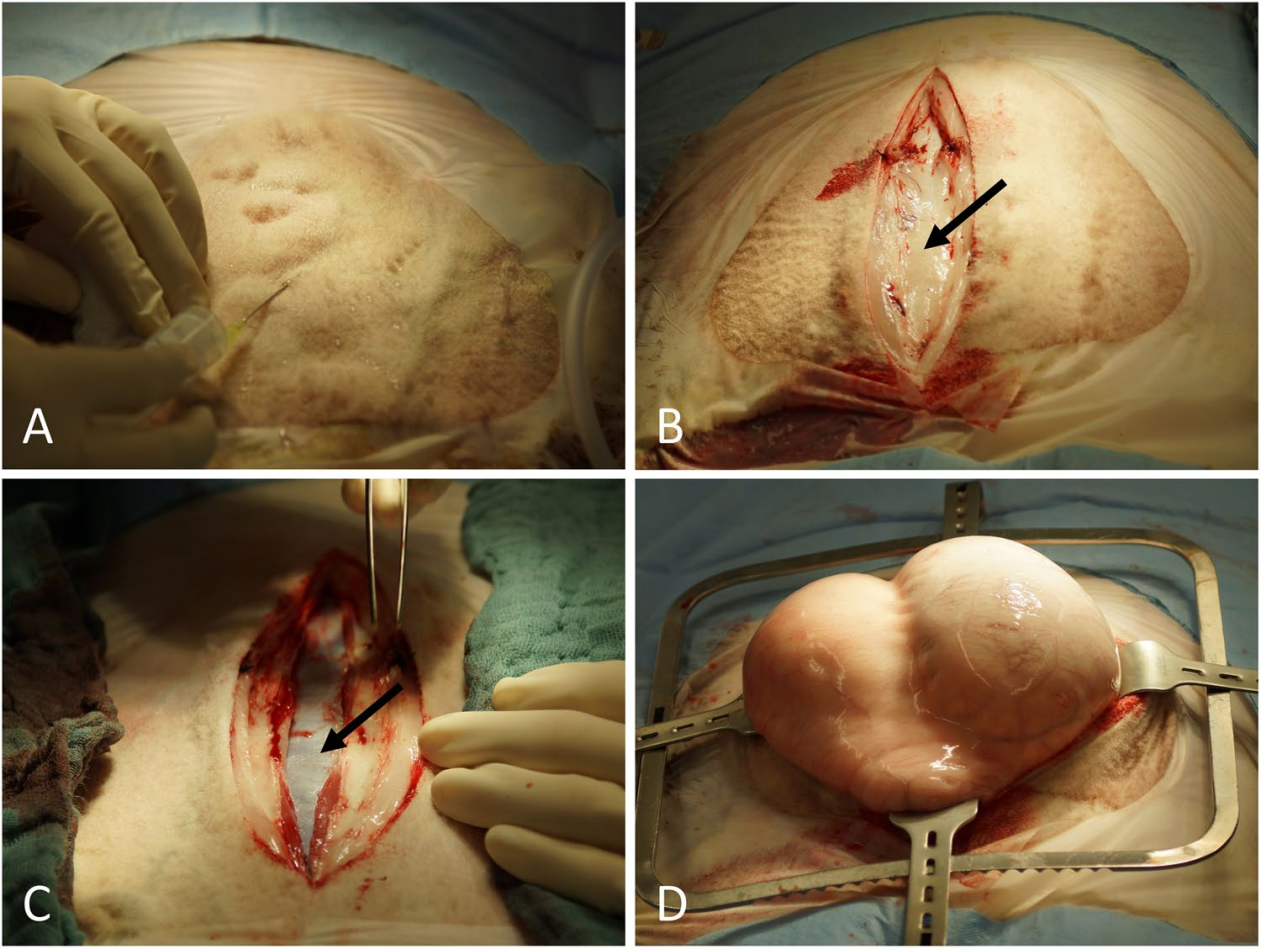
Intraoperative steps to access the uterus. **A** Line block with procainhydrochloride/epinephrine. **B** Subcutaneous tissue after skin incision (arrow). **C** Peritoneum (arrow) after sharp incision of the abdominal fascia and the muscular layers. **D** The part of the uterus carrying the fetus is brought out of the abdominal incision to enable controlled incision of the uterine wall and an uncomplicated extraction of the lambs

For surgery, ewes were placed in supine position on an operation table with high sides and ropes to fix the extremities to prevent dislocation of the animal during the operation (Figure 1B). Fur in the operating field was shorn (Figure 1C). The operation field was washed several times and disinfected with Braunoderm (B. Braun Medical, Sempach, Switzerland) followed by Octeniderm (Schülke & Mayr, Norderstedt, Germany). The whole animal was covered with a sterile drape with side collection bags for the amniotic fluid (Medline International Germany, Kleve, Germany). A paramedian line block was performed with 15 ml Isocain (20 mg/ml procaine + 0.025 mg/ml epinephrine, Selectavet, Weyarn, Germany) (Figure 2A). A paramedian abdominal incision of approximately 15 cm was performed while sparing the subcutaneous veins draining blood from the udder (Figs. 1D and 2B).

**Fig. 3:**
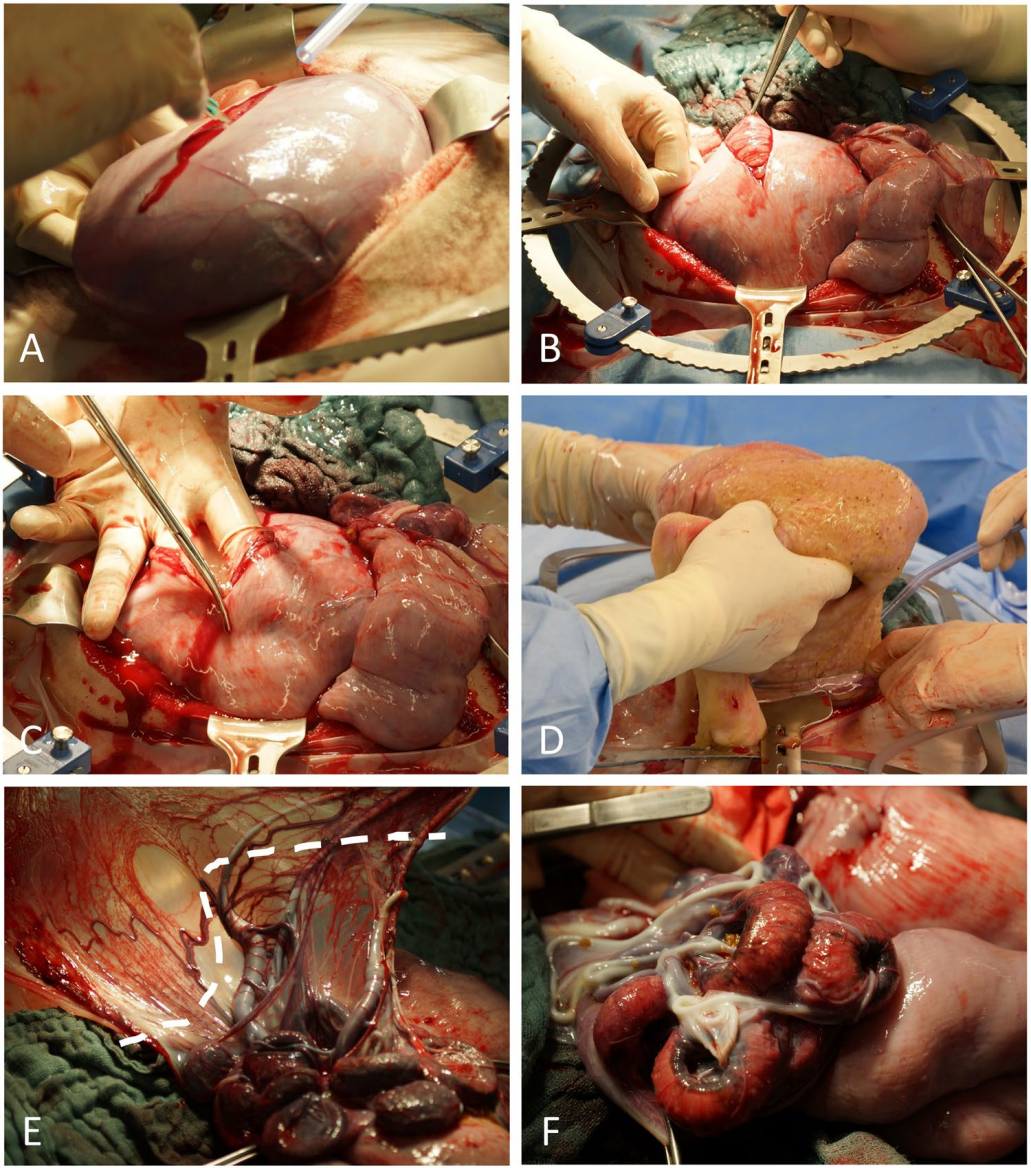
Extraction of lambs and excision of amniotic membranes. **A** Placentome-sparing incision of the uterine wall with a scalpel. **B** Presentation of the fetal membranes with tweezers membranes before opening with scissors. **C** Enlarging the hysterotomy with scissors while sparing placentomes. **D** Extraction of a lamb. **E** Amniotic membranes are excised generously while sparing large intraamniotic blood vessels and placentomes. This step is performed to leave as little fetal tissue as possible inside the uterus to help uterine involution and uncomplicated afterbirth. **F** Ovine placentomes. As the fetal and maternal part of the placentomes do not separate spontaneously during cesarean delivery, intraoperative removal of the syndesmochorial placenta is not possible

**Fig. 4:**
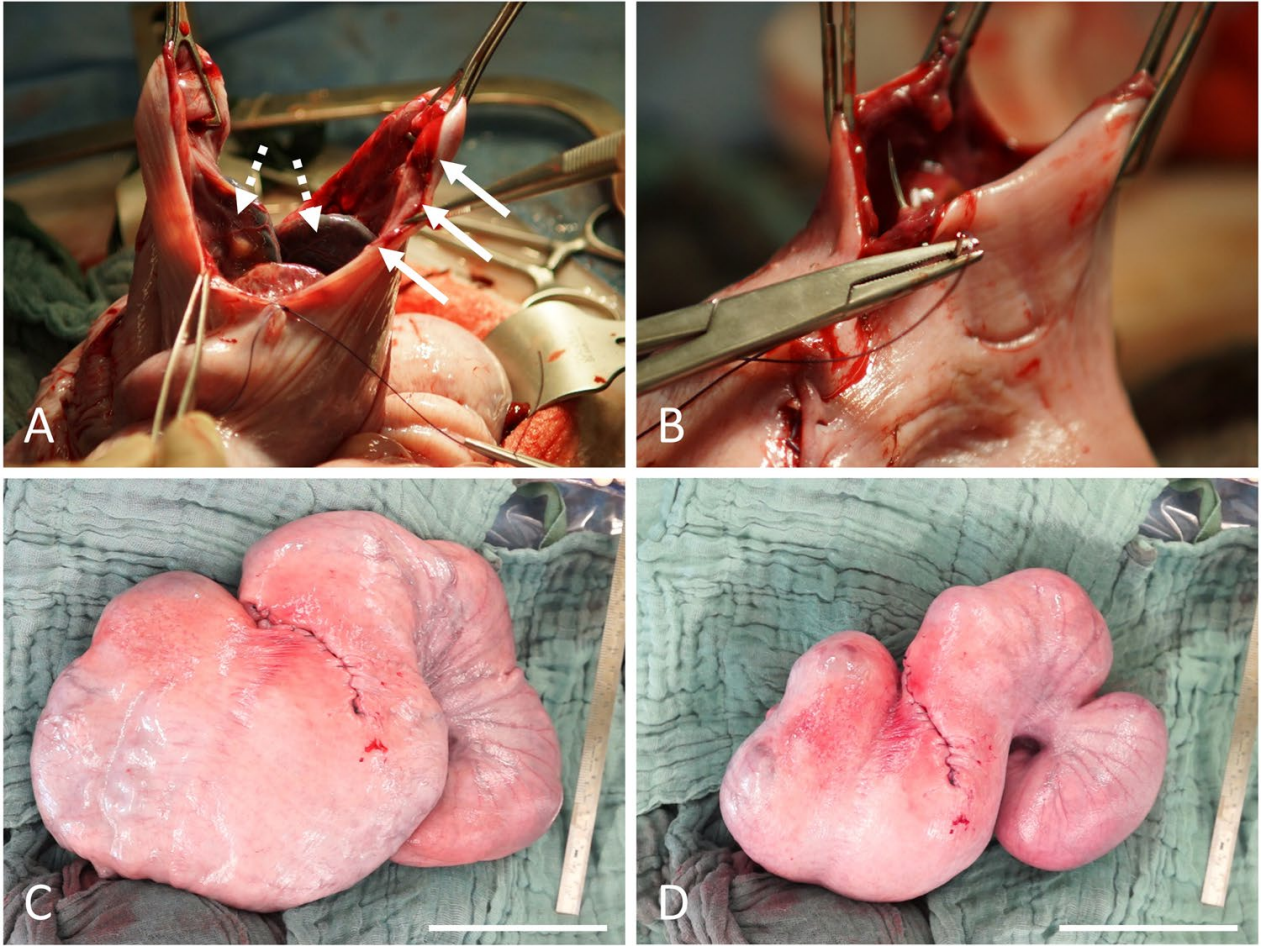
Intraoperative aspects of the ovine uterus after lamb extraction. **A** Exemplary view of the ovine uterine wall. The myometrium is ubiquitously 1–2 mm thick (solid arrows). In the uterine cavity, placentomes can be seen (dashed arrows). **B** Closure of the hysterotomy with continuous sutures. **C** Uncontracted uterus. **D** Strongly contracted uterus after IV administration of 3 IU oxytocin. Scale bars = 10 cm

The muscles of the abdominal wall and the peritoneum were carefully opened sharply, avoiding any injuries to the omentum (Figure 2C). The omentum was plugged upwards with lap sponges to get free access to the uterus. The uterine horn carrying the fetus was exteriorized to enable controlled incision of the uterine wall and an uncomplicated extraction of the lambs (Figure 2D). Sharp opening of the uterine horn in the large curvature without violating placentomes followed by gentle developing of the lambs (Figure 3A–D). In cases of multiple pregnancies where extraction of all lambs was not possible through one single hysterotomy, a second incision was performed in the other uterine horn. Thorough internal as well as external palpation ensured that no lambs remained in utero. Amniotic membranes were excised generously while sparing large intraamniotic blood vessels and placentomes to support uterine involution and uncomplicated afterbirth (Figure 3E). As the fetal and maternal part of the placentomes do not separate spontaneously during CD, the syndesmochorial placenta was left in situ (Figure 3F). Both horns were carefully milked out to remove as much amniotic fluid as possible. Closure of the uterus was performed with polyglactin sutures (0 CT-1 plus Vicryl, Johnson & Johnson, Norderstedt, Germany) (Figure 4A, B). Sodium hydrochloride 500 ml volume (Serumwerk Bernburg, Bernburg, Germany) was poured over the uterus to remove blood, other fluids, and tissue particles. Oxytocin was administered as described above to support uterine contraction (Figure 4).

**Fig. 5:**
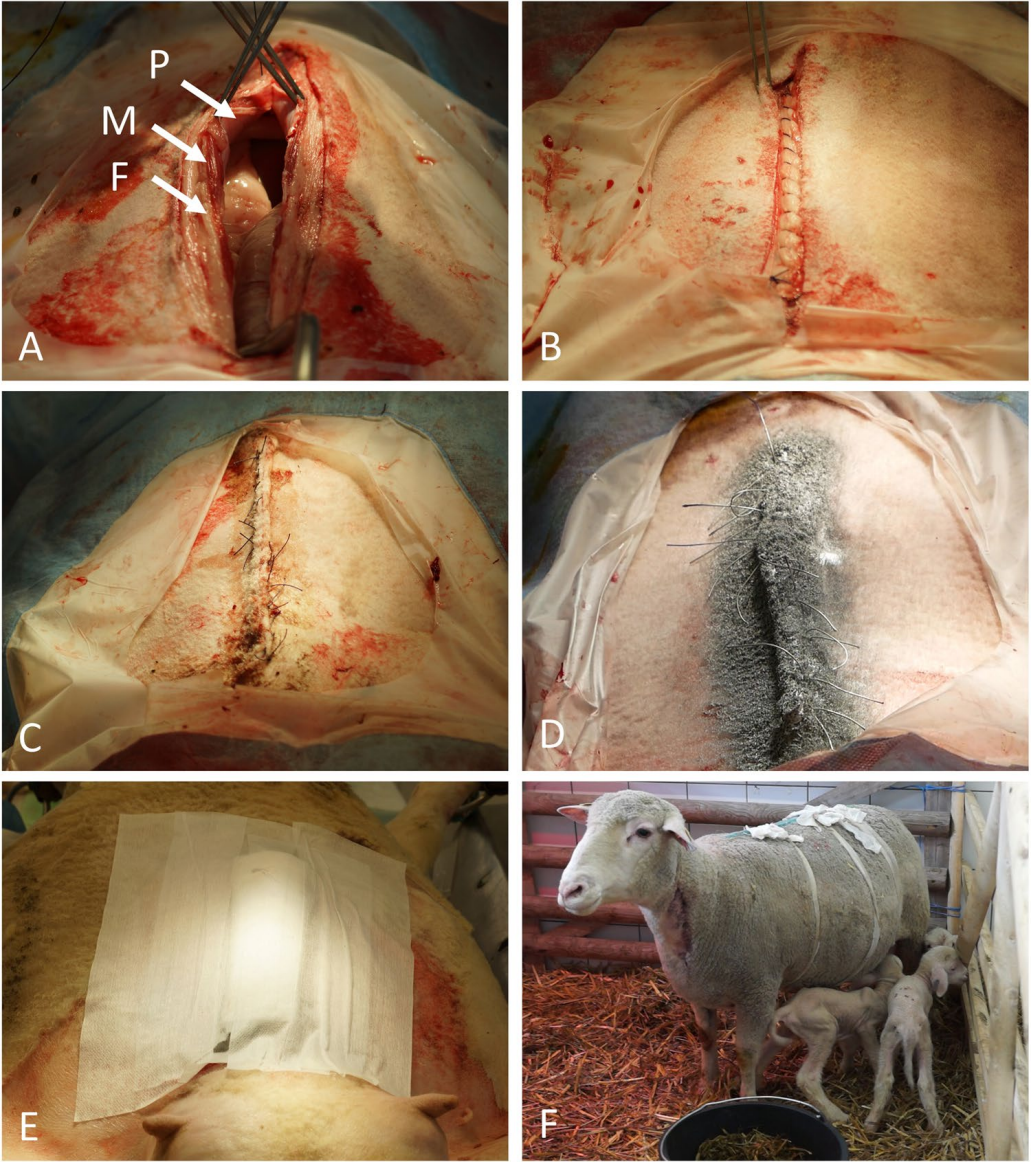
Closing the ovine abdomen and wound dressing system. **A** Abdominal wall before closure with continuous sutures. P, peritoneum; M, muscular layers, F, fascia. **B** View on the subcutaneous tissue that has been closed with continuous sutures. **C** Skin closed with mattress sutures. **D** Aluminum spray is applied on the wound to protect against contamination. **E** Dressing on the abdominal wound. **F** Three gazes are tied around the body to keep the wound dressing in place, ewes are kept in individual pens with their offspring to promote recovery and suckling of lambs

For animals 1–31, peritoneum, abdominal wall musculature, and fascia were closed with continuous polyglactin sutures (1 CTX plus Vicryl, Johnson & Johnson, Norderstedt, Germany) (Figure 5A). For animals 32–48, abdominal wall closure was modified according to a previously published method [27]: peritoneum, abdominal wall musculature, and fascia were closed with U-stitches using non-absorbable polyamid sutures (Supramid black, USP 7, SMI AG, Steinerberg, Belgium). The resulting bead of tissue was then fixed to the abdominal wall by means of a continuous suture with (Surgicryl, polyglycolic acid, USP 5, SMI AG). For all animals, a subcutaneous continuous suture was placed, and mattress sutures served for skin closure (both polyglactin, 1 CTX plus Vicryl) (Figure 5B, C). Afterwards, silver nitrate spray was applied on the wound and a self-adhesive bandage placed, supported by three gauze bandages tied around the ewe’s torso (Figure 5D–F).

### Ewes: Postoperative Care and Medication

Ewes were held in individual pens with their lambs for 11 days after CD and examined daily by a veterinarian to ensure that any distress or complications were addressed in a timely manner. During this period, respiration, behavior, food intake, defecation, body temperature, and the appearance of the wound and udder were monitored up to three times daily. Antibiotic prophylaxis was performed with amoxicillin (Zoetis Schweiz, Delémont, Suisse) 10 mg/kg s.c. for 3–10 days postoperatively. The analgesic meloxicam (Ceva Santé Animal, Libourne, France) 0.5 mg/kg s.c. was given on days 1 and 3 postoperatively and on other days only if required. Oxytocin 10 IU SC was given on days 0–3 postoperatively to support uterine contraction and timely afterbirth. A transabdominal and/or transrectal ultrasound to check uterine involution was performed 7–10 days after CD. The skin sutures were removed on postoperative day 10 (Supplementary Figure 1). A blood count and serum infectious parameters were determined immediately before CD and on the first postoperative day. Humane end points were defined as follows: weight deficit ≥ 20% compared to healthy animals of the same strain and age, sunken cloudy eyes, blood at body orifices, bloody feces, diarrhea (if debilitating or persistent), staggering/apathy, paresis/eclampsia/seizures, abscesses/purulent sores (no improvement with therapy within 36 h), and automutilation. All ewes survived without any major complications, the end points were not reached.

### Lambs: Neonatal Care and Feeding

**Fig. 6:**
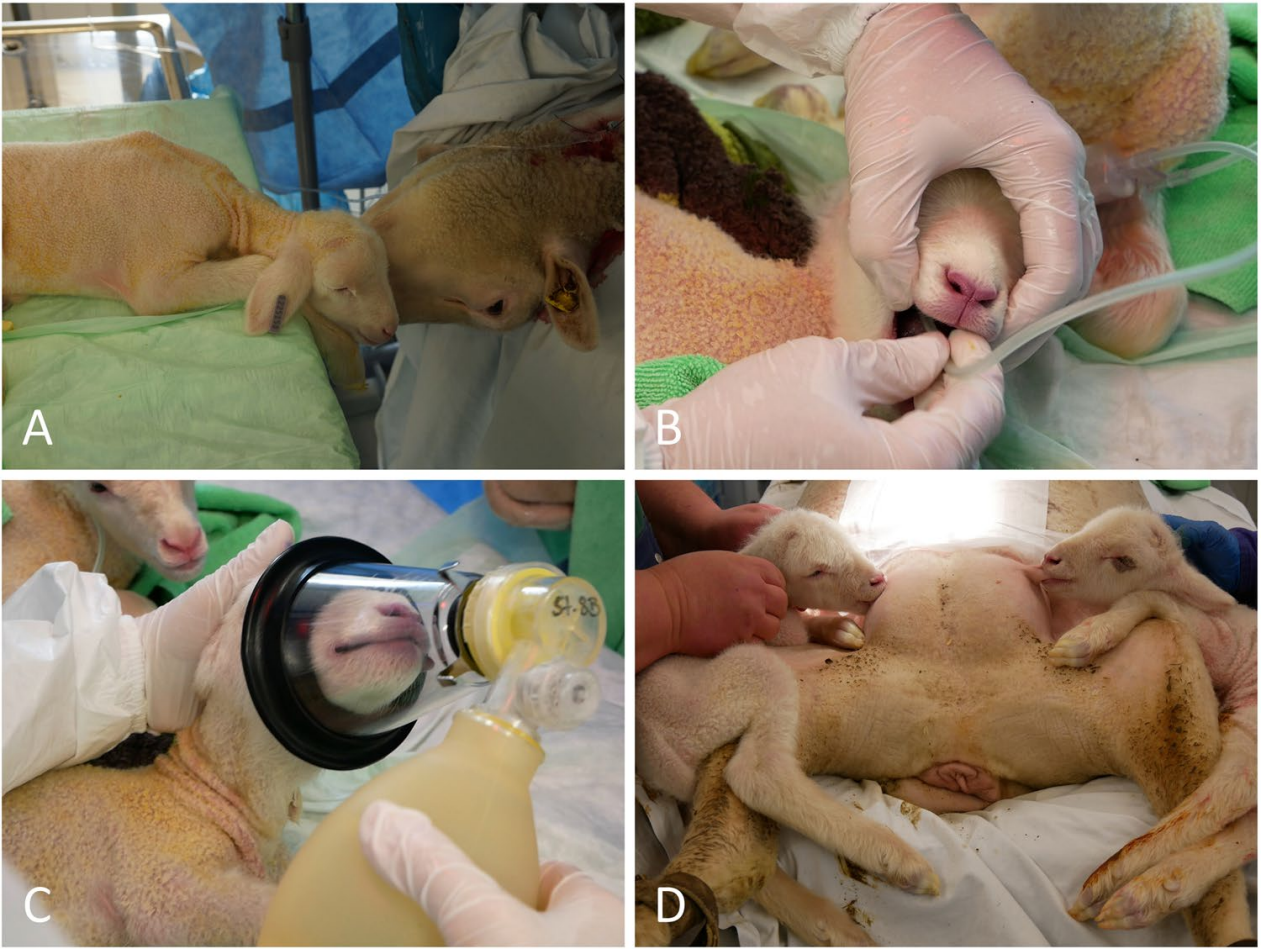
Neonatal lamb care. **A** Immediately after birth, lambs are positioned next to their mother’s head to promote ewe’s acceptance of the lambs. **B** Suction of amniotic fluid from the neonatal mouth, nose, and throat. **C** Lambs that show breathing difficulties are ventilated with a mask. **D** Immediately after cesarean delivery, lambs are encouraged to drink colostrum

Immediately after birth, the lambs were placed at the ewe’s head on a padded table under a heat lamp and dried with towels (Figure 6A). Amniotic fluid residues were suctioned from the mouth and muzzle (Figure 6B). In case of insufficient self-breathing after stimulation, ventilation was performed with a mask (Figure 6C). All lambs received 10 mg doxapram sublingually (Dechra Veterinary Products, Aulendorf, Germany). After CD, the lambs were allowed to drink colostrum from the sheep’s udder in the operating room before relocation of the sheep (Figure 6D). All lambs received 1500 IU SC anti-tetanus serum (WDT, Garbsen, Germany), the umbilicus was disinfected with iodine solution. Beginning on the day of delivery, lambs were encouraged actively to drink milk from their mother several times per day. For lambs that did not receive enough milk from their mother during the first days, bottle feeding was supplied as long as needed.

### Housing and Husbandry

Ewes were kept in group housing on deep litter in the sheep pen until the cesarean delivery was performed. The operating room is in close proximity to the barn, so that the sheep are housed in their familiar surroundings throughout the study. Postoperatively, the ewes were housed individually in the barn for at least 11 days in individual lambing pens with their lambs. After that, they were kept in small groups (ewes with lambs) on straw bedding. The ewes were kept there with their lambs in groups on deep litter. During the growing season, grazing is provided. For environmental enrichment, straw and hay were provided.

### Statistical Analysis

Sample size calculation was performed with the software nQuery + nTerim 3.0 [28]. As the underlying research question is concerned with uterine wound healing, the calculation is based on a publication that compares the ultrasonographic aspect of uterine scars in humans. In this study, 36 women were randomized into two groups and underwent closure of the hysterotomy by single-layer or double-layer suture during elective cesarean delivery. The primary outcome measure is the thickness of the lower uterine segment in mm on transvaginal ultrasound (hydrosonography) 6 months after CD [29]. Analysis of variance showed that to detect differences between four groups, at least 11 samples per group would be needed (*α* = 0.05, power = 0.80, effect size = 0,28). Therefore, 12 animals were included per group (4 × 12 = 48). Data are reported as median and interquartile range, categorical variables are expressed as numbers and percentage. SPSS Version 28 (IBM, Chicago, USA) was used for statistical analysis.

## Results

### Management and Timing of CD

All 48 pregnant ewes received CD at 142–144 dG according to our protocol. No ewe delivered prematurely or showed signs of approaching birth. Pregnancy toxemia did not occur.

### Intraoperative Findings and Ovine Anatomy

**Fig. 7:**
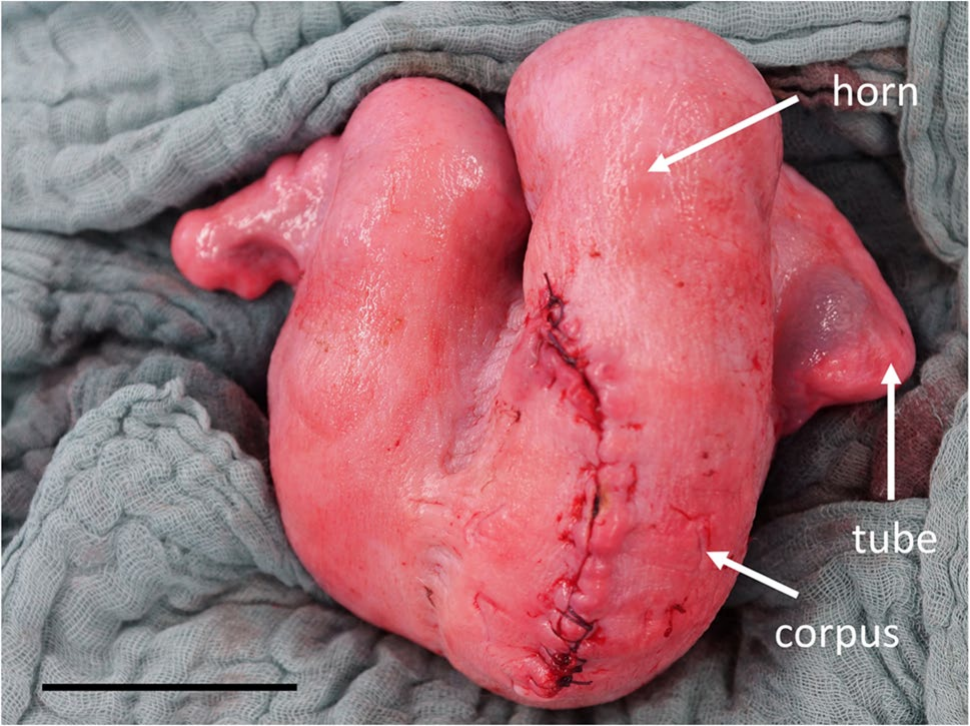
Ovine intraoperative uterine anatomy. Uterine corpus, horns, and tubes are shown. Note that the incision has been performed in the uterine corpus and not in the horn

All essential steps were documented by video (Video 1). Upon entry into the abdominal cavity via paramedian incision, the uterus was identified immediately. It was difficult to determine which horn was seen due to limited space to explore the abdominal cavity with the fetus(es) still in utero. The fetus-carrying horn was brought near the abdominal wall and a hysterotomy of 10–15 cm was performed (Figure 3A). Exteriorizing the uterus in front of the abdominal wall was only feasible after extraction of all lambs. Only then was it clearly possible to evaluate the whole uterine anatomy (Figure 7). Incising the uterine corpus with the aim to extract the lambs from there was not always feasible due to limited space in the ovine situs with the lambs still in utero. The myometrium both in the uterine corpus and cornua was ubiquitously 1–2 mm thick before administration of oxytocin and 2–3 mm afterwards (Figure 4A, B). Removing the perimetrium from the myometrium as during bladder-flap formation in human CD was not possible due to the thin uterine wall in the cornua uteri. As in some regions, the placentomes were located 5–10 cm away from each other, they were easily avoided during hysterotomy. After birth of the lambs, placentomes were not removed to avoid excessive bleeding and endometrial damage that might prevent future pregnancies. Suturing the uterine wall proved possible with different techniques also practiced during human CD: single-layer locked suture, single-layer continuous suture, double-layer continuous suture, or interrupted sutures. Due to non-separation of the placenta, estimated blood loss was minimal (< 100 ml/ewe). Due to the contraction-free state of the ewes and the overall thin myometrium, the uterus appeared completely atonic after delivery of the lambs. Excising as much of the amniotic membranes as possible while milking out the remaining amniotic fluid proved to be a feasible way to aid uterine contraction. Suturing the abdominal wall was uneventful as described in the methods section.

### Maternal and Neonatal Outcomes

All ewes survived. Due to regional anesthesia, all animals were able to stand, walk, and eat immediately after the operation.

Delivery of the placenta was observed in 28 ewes. It occurred after 2 (IQR: 1–4, range: 0–21) days. In all other animals, complete uterine involution was observed with ultrasound at latest 3 weeks after CD, so that the placenta must have been expelled in time. No cases of metritis or wound infection occurred. All ewes started lactating. Mastitis was diagnosed in three ewes and treated with local antibiotics. One ewe presented with asymptomatic scar hernia with intact skin and inconspicuous general condition in the first postoperative weeks. Minimal intraoperative blood loss was confirmed by similar preoperative and postoperative hemoglobin levels (Table 1). A total of 87 lambs were born with five having already died in utero. The macerated fetuses with thrombosed umbilical vessels did not show evidence of infectious cause of death or any notifiable animal disease. Five lambs died postnatally (3 in the first hour, one on the second day, one on the third day), most probably due to immaturity. Five lambs were born clearly underdeveloped and not viable from two ewes (#28, #31) despite secured mating date. They were euthanized immediately after birth. In the further course of the study, gestational age was verified sonographically on the basis of placentome size before each cesarean delivery [30, 31]. The 77 living lambs had uneventful postnatal courses with adequate weight gain. Twenty-five lambs were exclusively fed by their mother from the beginning while 45 received additional bottle feeding for 3 (2–9) days. Three lambs were not accepted by their mother but were fed by another ewe. Four lambs were not fed by any ewe and were therefore exclusively bottle-fed.

**Table 1:** Maternal baseline characteristics, birth, and postpartum details

	Merino ewes (*N* = 48)
Baseline characteristics	
Maternal age in years*	5 (2–6)
Weight before delivery in kg*	87 (76–94)
Previous pregnancies*	2 (1–4)
Birth	
Gestational age at delivery^†,‡^	
142 days	11 (23.9)
143 days	34 (73.9)
144 days	1 (2.2)
Onset of labor and/or ruptured membranes^†^	0
Lambs per ewe^†^	
One	13 (27.1)
Two	27 (56.3)
Three	8 (16.7)
Total number of lambs^†^	91 (100)
Live-born lambs	87 (95.6)
Postnatally deceased lambs	5 (5.7)
Euthanized lambs^¶^	5 (5.7)
Dead-born lambs/miscarriage	4 (4.4)
Sex of live-born lambs^†,§^	
Female	39 (44.8)
Male	48 (55.2)
Postpartum details	
Metritis^†^	0 (0)
Mastitis^†^	3 (6.3)
Birth of the placenta (in days postpartum)*^,‡^	2 (1–4)
Wound infections	0 (0)
Preoperative hemoglobin level (mmol/l)*	5.4 (4.8–5.8)
Postoperative hemoglobin level (mmol/l)*	6.0 (5.1–6.5)
Other morbidities requiring specific therapy^†^	One abdominal scar hernia (4.3)

*Data presented as median (IQR)

^†^Data presented as *N* (%)

^‡^*N* = 46 as two ewes have been excluded as they gave birth to lambs clearly underdeveloped and not viable (despite secured mating date)

^¶^Five lambs were born clearly underdeveloped and not viable, see also ^‡^

^§^*N* = 28 as in 20 sheep placental birth was not observed. However, the uterus appeared empty in postoperative ultrasound 2–3 weeks after cesarean delivery

## Discussion

CD can be safely performed in sheep with little blood loss and few postoperative complications. For an optimal view of the uterus, paramedian abdominal incision seems more convenient than a left flank incision, which is more often used by veterinarians. The paramedian approach and technique presented appear safe as we did not experience any wound infections. For research questions such as uterine wound healing, the ovine anatomy is comparable to human anatomy. However, the myometrium of the ewe is generally thinner (1–2 mm) than in the human (5.4–10.7 mm) [32]. Furthermore, it was not always possible to primarily incise the corpus of the bicornuate ovine uterus for extraction of the fetus, as it is located deeply in the pelvis behind the udder, so that hysterotomy was often performed in the greater curvature of the uterine horn. This is a major difference of CD in humans, where hysterotomy is usually performed in the lower uterine segment [33].

In contrast to human beings, the uterus of all ewes appeared atonic after delivery of the neonates. However, only little bleeding occurred, probably because the fetal membranes and placentomes do not separate from the uterine wall. Interestingly, retention of fetal membranes and its possible complications did not represent a major health issue.

This study complements the existing literature on CD in pregnant sheep, as it evaluates the translatability of ovine uterine anatomy and CD techniques to humans. Veterinary publications mainly describe surgical approaches with limited information needed for translational surgical sheep models [17, 19, 21]. For example, veterinarians routinely perform CD through an incision in the left sublumbar fossa [19]. However, this makes it more difficult to investigate the whole uterus compared to paramedian laparotomy. In contrast, sheep models used to answer research questions for humans often did not focus on animal survival. Even in studies with a neonatal focus, lambs and/or ewes have often been sacrificed [3, 15, 16, 34]. As a result, there is little detailed information on how to follow up sheep after a planned CD, which represents a hurdle for obstetric researchers in developing new sheep models while maintaining animal welfare. In the field of uterine wound healing after CD, only one small study has been published with eight non-pregnant ewes included. Biomechanical and pathological examinations of uterine scars after single- or double-layer closure during CD revealed larger fibrotic areas in the double-layer closure group without significant differences in biomechanical properties. Also in this study, newborn lambs were immediately sacrificed and ewes were sacrificed 8 months after CD [13]. Our study represents the first detailed description of a sustainable safe sheep model for CD, which is in line with contemporary ethical and animal welfare considerations due to the multidisciplinary team including veterinarians, shepherds, and animal keepers. The protocol described in this study can inform other research projects on how to perform CD in sheep while safeguarding animal welfare of both ewes and lambs. The study has shown that an immense effort in terms of personnel is needed to preserve the well-being of ewes and lambs. During CD, appropriately trained staff and material are vital for neonatal care of up to three lambs per ewe. Daily visits (up to three) of the sheep and 24 h availability of doctors for ~11 days post-CD are needed. Active support and close monitoring of ewe-lamb bonding were essential to keep the rate of maternal rejection low and thus minimize the number of lambs that need to be bottle-fed, a procedure that also requires a significant amount of personnel. We worked with six persons in the operation theater (two surgeons, one anesthesiologist, two nurses for neonatal care, one person for operating assistance and documentation) and 2–3 shifts of animal keepers in the pen to ensure continuous postoperative care.

Most publications on cesarean delivery in sheep provide little information on postoperative wound care after CD in sheep [19, 21, 35]. Phytian et al. have recently published a review of clinical experience and referral works in ovine cesarean deliveries [36]. The authors suggest assessing ewes daily for signs of pain and discomfort and monitoring skin wound healing for 10–14 days postoperatively, which is in line with our protocol. They further describe in detail how to manage moderate to major cesarean skin wound dehiscence, which mostly occurs during the first 1–2 weeks [36]. As no skin wound dehiscence appeared in this study, we have not elaborated on this topic.

At last, it needed to be planned what happens with the animals after the research project. When large numbers of both ewes and lambs survive, it needs to be clear where the animals can stay and whether they may be slaughtered. As not all drugs are approved for animals whose meat might be eaten, careful choice of the applied drugs is necessary. The establishment of this sustainable animal-friendly sheep model for CD enables further translational medicine research in various fields, such as uterine wound healing after different hysterotomy closure techniques or comparative studies on physiological and histological aspects of the uterine wall (endometrium, myometrium) of humans vs. sheep. To further improve the neonatal outcome, studies might address the optimal timing of delivery and dose of antenatal corticosteroids without risking unintended parturition. The study has several strengths. This study is the first to describe a detailed protocol for CD in sheep from a human medicine perspective. Its strength lies in the large sample size of 48 sheep that gives a representative picture of maternal and neonatal outcomes with less complications than described in the literature [35, 37]. Extensive photo and video documentation covers all the necessary details on the operation itself as well as peri- and postoperative management (Video 1). It therefore provides numerous practical details for future studies.

The study has some limitations. As described, one ewe experienced a scar hernia. One reason might be the paramedian entry into the abdominal cavity and the continuous suture with suture material that is commonly used in human beings. Although our protocol was adapted from available literature [20, 21, 38], another method would be the incision of the abdomen in the flank region where there is less pressure from the internal organs and abdominal wall muscles are thicker. Incisional hernias are therefore less frequent [35]. Alternatively, the abdominal wall can be closed with interrupted sutures or even thicker suture material. We resorted to this adaptation after experiencing two cases of scar hernia.

The macroscopic anatomy of the sheep differs from human anatomy due to the presence of a bicornuate uterus. In humans, this anatomical feature is present in only two out of 1000 females [39]. It can therefore be debated which surgical aspects are comparable, as in sheep the hysterotomy is generally performed in the region of the large curvature of the uterine horns [19]. As the lower uterine segment is located behind the udder, it cannot be incised primarily. Furthermore, two ewes gave birth to lambs clearly underdeveloped and not viable (despite secured mating date). These five lambs were euthanized immediately after birth. In the further course of the study, gestational age was verified sonographically on the basis of placentome size before each cesarean delivery [30, 31]. At last, our protocol to routinely administer oxytocin, prostaglandins, and antibiotics both intra- and postoperatively has been designed to minimize the risk of retained fetal membranes or infections and is not common in veterinary practice [20]. Whether the same favorable outcomes might be achieved also with less medication could be determined in the future.

## Conclusion

This sheep model constitutes a feasible and safe approach to evaluate anatomical and surgical aspects of CD in contraction-free ewes from a human medical perspective. The risk of adverse events such as pregnancy toxemia, wound infections, placental retention, endomyometritis, or not-acceptance of the lambs by their mothers is minimal. With adequate planning and a reasonable number of staff, it is possible to safeguard both maternal and neonatal survival and thus to adhere to modern animal welfare laws. This model can be used for studies to improve uterine wound healing both after previous CD and fetal surgery—two aspects highly important for clinical obstetrics. This sustainable translational medicine model offers additional potential for the offspring to be used for further research studies (e.g., transgenerational inheritance research).

### Supplementary Information

ESM 1 Video 1 Cesarean delivery of a pregnant ewe at 143 days of gestation (MP4 92.2 mb)

ESM 2 Supplementary Fig. 1 Laparotomy wound of a sheep on postoperative day 10 after removal of skin sutures (TIF 97675 kb)
